# Antimicrobial and Antibiofilm Activities of New Synthesized Silver Ultra-NanoClusters (SUNCs) Against *Helicobacter pylori*

**DOI:** 10.3389/fmicb.2020.01705

**Published:** 2020-07-31

**Authors:** Rossella Grande, Francesca Sisto, Valentina Puca, Simone Carradori, Maurizio Ronci, Antonio Aceto, Raffaella Muraro, Gabriella Mincione, Luca Scotti

**Affiliations:** ^1^Department of Pharmacy, “G. d’Annunzio” University of Chieti–Pescara, Chieti, Italy; ^2^Center for Advanced Studies and Technology (CAST), “G. d’Annunzio” University of Chieti–Pescara, Chieti, Italy; ^3^Department of Biomedical, Surgical and Dental Sciences, University of Milan, Milan, Italy; ^4^Department of Medicine and Aging Science, “G. d’Annunzio” University of Chieti–Pescara, Chieti, Italy; ^5^Department of Medical, Oral, and Biotechnological Sciences, “G. d’Annunzio” University of Chieti–Pescara, Chieti, Italy

**Keywords:** *Helicobacter pylori*, drug resistance, silver nanocluster, antibiofilm activity, FIC index

## Abstract

*Helicobacter pylori* colonizes approximately 50% of the world’s population, and it is the cause of chronic gastritis, peptic ulcer disease, and gastric cancer. The increase of antibiotic resistance is one of the biggest challenges of our century due to its constant increase. In order to identify an alternative or adjuvant strategy to the standard antibiotic therapy, the *in vitro* activity of newly synthesized Silver Ultra-NanoClusters (SUNCs), characterized by an average size inferior to 5 nm, against clinical strains of *H. pylori*, with different antibiotic susceptibilities, was evaluated in this study. MICs and MBCs were determined by the broth microdilution method, whereas the effect of drug combinations was determined by the checkerboard assay. The Minimum Biofilm Eradication Concentration (MBEC) was measured using AlamarBlue (AB) assay and colony-forming unit (CFU) counts. The cytotoxicity was evaluated by performing the MTT assay on the AGS cell line. The inhibitory activity was expressed in terms of bacteriostatic and bactericidal potential, with MIC_50_, MIC_90_, and MBC_50_ of 0.33 mg/L against planktonic *H. pylori* strains. Using the fractional inhibitory concentration index (FICI), SUNCs showed potential synergism with metronidazole and clarithromycin. The biofilm eradication was obtained after treatment with 2×, 3×, and 4× MIC values. Moreover, SUNCs showed low toxicity on human cells and were effective in eradicating a mature biofilm produced by *H. pylori*. The data presented in this study demonstrate that SUNCs could represent a novel strategy for the treatment of *H. pylori* infections either alone or in combination with metronidazole.

## Introduction

*Helicobacter pylori* (*H. pylori*) colonizes approximately 50% of the world’s population, with a wide variation in the prevalence of the microorganism among regions and countries (Hooi et al., 2017). *H. pylori* is associated with the development of chronic gastritis and peptic ulcer disease (Eshraghian, 2014), and in 1994, it was classified as type one carcinogen (International Agency for Research on Cancer (IARC) 2019). Epidemiological studies indicate that *H. pylori* eradication is associated with a decrease of incidence of gastric cancer (Kosunen et al., 2011) that represents the third most common cancer worldwide with a survival rate longer than 5 years after the diagnosis in 1 over 5 patients affected by this malignancy (Grande et al., 2010; Hooi et al., 2017). The failure of the antibiotic treatment may be due to several factors such as improper regimen, poor patient compliance, internalizing bacteria, gene mutations, transfer of resistance genes, and biofilm formation (Hu et al., 2017). *H. pylori* has been defined as a “quasi-species” for its wide genetic variability. In fact, it has been demonstrated that *H. pylori* strains isolated from different patients displayed a high degree of variability for the acquisition of new DNA sequences (Taylor et al., 1992; Suerbaum, 2000; Grande et al., 2010). Many studies showed the co-colonization in the same patient of multiple *H. pylori* strains characterized by the resistance or susceptibility to the same antibiotic. Recombination events can occur in the same patient during *H. pylori* infection due to the adaptation of a single strain to stressful stimuli such as sub-inhibitory antibiotic concentrations as well as to mixed infections deriving from the host colonization by different *H. pylori* strains in time (Grande et al., 2010). In addition, *H. pylori* can produce a well-structured biofilm characterized by the presence of outer membrane vesicles (OMVs) associated with extracellular DNA (eDNA) (Cole et al., 2004; Cellini et al., 2005; Yonezawa et al., 2009; Grande et al., 2015). It has been demonstrated that the eDNA associated with OMVs facilitates cell-to-cell binding and contributes to the stability of the biofilm matrix. Moreover, the nucleic acid is protected by the vesicle structures guaranteeing both delivery and release of the genetic information and important proteins in other bacterial cells, and *H. pylori* pathogenesis and survival (Grande et al., 2015; Ronci et al., 2019). In addition, Yonezawa et al. (2011) demonstrated the key role of a 22-kDa protein delivered by OMVs in the development of the biofilm of *H. pylori* TK1402. A biofilm can be defined as a complex tridimensional structure in which cellular aggregates are immersed in a self-produced matrix of extracellular polymeric substances (EPS) (Flemming et al., 2016). Biofilm formation is a multistep regulated process in which cellular adherence, EPS secretion and detachment of bacteria from the maturing biofilm, are controlled by the regulation of several genes (Grande et al., 2014).

*Helicobacter pylori* cells aggregation and biofilm formation guarantee antibiotic tolerance versus the drugs commonly used in anti-*Helicobacter* therapy as well as the protection from the host immune system. The variability in the composition and structure of *H. pylori* biofilm suggests the use of multi-targeted or combination therapies. In fact, the tolerance against the antimicrobial drugs might be due to both the slow penetration of drugs through the EPS matrix and the presence of viable but not culturable (VBNC) cells characterized by a metabolic dormancy (Sisto et al., 2000; Dakal et al., 2016). Therefore, the evaluation of *H. pylori* strain susceptibility versus the antimicrobial drugs *in vitro*, traditionally carried out only on the planktonic phenotype, does not represent a reliable predictor of the efficacy of the antimicrobials in the human stomach (Yonezawa et al., 2015). In fact, Yonezawa et al. (2015) and Yonezawa et al. (2013) demonstrated that *H. pylori* biofilm development increased the resistance to clarithromycin at MIC levels by up to 4-fold in 2-day biofilms and to 16-fold in 3-day biofilms; thus, *H. pylori* biofilm phenotype induces the generation of clarithromycin resistance mutations. In conclusion, as previously reported by Yonezawa et al. (2015), the study of new *H. pylori* eradication strategies using biofilm-dissolving molecules or compounds may provide advantages in resolving *H. pylori* infections.

For the last few years, the antibiotic resistance represents one of the biggest problems of global health due to the constant increase. More than 50 antibiotics are currently under clinical development, but only a few of them are innovative and can get to the market. The prophylaxis by vaccination is also under preclinical or clinical investigation, but licensed vaccines for *H. pylori* infection are not yet available. Therefore, the therapy improvement remains the only way to fight this infectious disease.

The triple therapy, consisting of a proton-pump inhibitor (PPI) and two different antimicrobials, has been considered the standard therapy for the *H. pylori* eradication for the last 20 years. The increase of the failure rates of the triple therapy recorded in many countries such as those in Europe, as well as Korea, Japan, and China, has been due to an excessive use of antibiotics as well as to the empirical prescription of drugs (Ko et al., 2019). Therefore, both a bismuth-containing quadruple therapy has been recently recommended or clinical studies involving novel potassium-competitive acid blockers (P-CABs instead of PPIs) have been carried out (Mori and Suzuki, 2019) as the first-line treatment of multidrug-resistant *H. pylori* strains, particularly in areas of high clarithromycin resistance (Macías-García et al., 2019). In 2017, the WHO classified the *H. pylori* resistance to clarithromycin as “a high priority for antibiotic research and development” (Savoldi et al., 2018). The resistance rate to clarithromycin is 30% in Italy and Japan, and 40% and 50% in Turkey and China, respectively (Yonezawa et al., 2013; Ghotaslou et al., 2015; Kocazeybek and Tokman, 2016; Kuo et al., 2017). A new approach should evaluate the use of alternative therapies that does not induce resistance and are effective on sensitive or resistant strain as well.

Based on these considerations, it is necessary to identify an alternative or adjuvant strategy to the standard antibiotic therapy, in order to reduce the development of any antibiotic resistance. Many recent studies report the antimicrobial efficacy of metallic nanomaterials, suggesting their potential use in medical devices, burn dressing, water treatment, and food preservation (Vimbela et al., 2017). Among all types of nanoparticles developed and characterized, silver nanoparticles (AgNPs) gained strong attention due to their inherent characteristic of acting as an antimicrobial agent even in solid state. AgNPs showed a strong antimicrobial activity against several Gram-positive and Gram-negative microorganisms as well as an antibiofilm effect (Sondi and Salopek-Sondi, 2004; Jain et al., 2009; Puca et al., 2019).

More in detail, AgNPs showed to be effective against *H. pylori* strains at very low concentrations (Amin et al., 2012, 2014; Gurunathan et al., 2015; Saravanakumar et al., 2019). They have also been tested on a large number of continuous and immortalized human cell lines in order to define their cytotoxic effect. However, it is worth noting that, due to the source variability and the differences in chemical–physical properties of the nanoparticles used in several studies, the results obtained are not easily comparable. Another important feature of many phytogenic or chemically synthesized types of AgNPs is the presence of surfactants, antioxidants, and/or organic stabilizers (Mathur et al., 2018; Shanmuganathan et al., 2019). To overcome these issues, we have recently synthesized novel Silver Ultra-NanoClusters (SUNCs) (patent pending EP-18181873.3) using a reproducible electrochemical approach. In contrast to conventional AgNPs, our silver solutions are stable in ultrapure water (UPW) and do not contain any surfactant or organic stabilizer. Moreover, due to their exceptional nanosize (average less than 5 nm), we named them SUNCs. Several studies indicate that small nanoparticles induce greater cytotoxicity than larger ones (Duran et al., 2010; Riaz et al., 2017; Siddiqi et al., 2018). The smaller size of nanoparticles is related to greater surface area that promotes higher agglomeration around the cell wall with consequent membrane damage (Lu et al., 2019). In general, the higher cytotoxicity of smaller nanoparticles can be explained by different chemical–physical properties occurring when the particle is formed by a lower aggregation of atoms. As an example, we can consider the observed increased ROS production of small nanoparticles as compared to that generated by the greater ones (Hossain et al., 2019). Other mechanisms associated with these silver-containing nanosystems are the disruption of the bacterial membrane, uncoupling of the respiratory electron transport, morphological changes (cytoplasm membrane detachment), DNA replication impairment, and protein expression alteration (Durán et al., 2016; Tang and Zheng, 2018). The aim of the present study was the evaluation of the antimicrobial and antibiofilm activity of these newly synthesized SUNCs, alone and in association, against *H. pylori* strains characterized by a different antibiotic susceptibility pattern. In addition, their cytotoxic effect on AGS cells was also investigated. This is the first report dealing with silver nanosystems endowed with an average dimension inferior to 5 nm in order to comprehend the further preclinical applications *in vivo*.

## Materials and Methods

### Bacterial Strains and Cell Culture

Eight clinical isolates of *H. pylori*, including two highly resistant to metronidazole (MNZ) (MIC > 8 mg/L), two resistant to clarithromycin (CLR) (MIC > 0.5 mg/L), two resistant to both MNZ and CLR, and two CLR, MNZ, and amoxicillin (AMX) susceptible, were tested. The isolated strains have been previously used in other research studies (Brenciaglia et al., 1996; Sisto et al., 2000; Zengin et al., 2018). The study did not require ethical approval, because all the isolates were obtained as part of routine diagnostic microbiology investigations; however, patients with duodenal ulcer or gastritis enrolled at the Microbiology Laboratory of “Luigi Sacco” – ASST Fatebenefratelli Sacco, Milan hospital, gave informed consent for further future studies.

A reference strain of *H. pylori* (ATCC 43504) was used as a control. Antimicrobial drugs were purchased from Sigma-Aldrich (St. Louis, MO, United States) and prepared according to the Clinical and Laboratory Standard Institute (Clinical and Laboratory Standard Institute [CLSI] 2007). The strains were cultured on Columbia agar base (Difco, BD, San Jose, CA, United States) supplemented with 10% (*v/v*) horse serum (Seromed, Biochrom, Germany) and 0.25% (*w/v*) bacto yeast extract (Difco, BD, San Jose, CA, United States). Plates were incubated for 72 h at 37°C under microaerophilic conditions (85% N_2_, 10% CO_2_, 5% O_2_).

Human gastric adenocarcinoma AGS ATCC CRL-1739^TM^ cell line (American Type Culture Collection, Manassas, VA, United States) was cultured in a 5% CO_2_ atmosphere in F-12 medium (HyClone, Logan, UT, United States) supplemented with 10% (*v/v*) decomplemented fetal bovine serum (FBS) (EuroClone SpA, Milan, Italy).

### Synthesis and Characterization of SUNCs

Silver Ultra-NanoClusters were electrochemically synthesized by means of an improved synthetic protocol in UPW without stabilizing agents or other chemical components as previously reported (Scotti et al., 2017). The novelty is based on an “ultra small” size without changing the complex set of physical and chemical properties (Z-Potential, Plasmonic UV-Vis absorbance, concentration, stability in acid pH). The method is protected by European Patent pending (EP-18181873.3). The product was a yellow solution with an absorbance maximum at 410 nm, odorless with a pH of 7–8, and characterized by good stability (6 months). No change in absorbance and λ_max_ was observed after 1 h of incubation of SUNCs at pH 3.5 in 0.01 M acetate buffer. A drastic decrease of the plasmonic spectrum absorbance, without change of the λ_max_, was observed after nitric acid treatment (0.1 M, pH < 1) for 10 min at 25°C. The complete disappearance of the SUNCs UV-Vis spectrum is obtained after 60 min of nitric acid (0.1 M final concentration) reaction at 60°C (Supplementary Figure S1; Ertürk, 2019). After each synthesis, large nanoclusters were removed by flushing the solution through 0.1-μm syringe filter devices (Whatman CYCLPR) and subsequent centrifugation at 13,023 × *g* for 15 min. SUNCs were characterized by transmission electron microscopy (TEM) in terms of concentration, shape, and size determination (75 kV ZEISS 109 equipped with Gatan-Orius SC200W-Model 830.10W TEM CCD Camera). Particle concentration was taken at 75 kV after evaporation of a drop of diluted SUNCs solution (1:5) on 300 mesh formvar-coated nickel grids and confirmed by ion-selective electrode (ISE) technique. Evidence of non-spherical shape was reported by TEM at 250,000× magnification, and the images were elaborated with ImageJ software (ImageJ bundled with Java 1.8.0_172).

Particle numbers and distribution were calculated using a statistical software Origin ver. 9.0; their good stability in the time (>1 year) was also verified (Scotti et al., 2017). The morphology and the elemental composition of the particles were analyzed by scanning electron microscopy (SEM) using a Phenom xl (Thermo Fisher Scientific, United States) equipped with BDS, SED, and EDS detectors (15 kW of acceleration voltages under high vacuum level).

### Antimicrobial Activity of SUNCs

Minimal inhibitory concentrations (MICs) and minimum bactericidal concentrations (MBCs) were determined by modified broth microdilution method as previously described (Sisto et al., 2009). Briefly, twofold serial dilutions of SUNCs were prepared in MegaCell^TM^ RPMI-1640 medium (Sigma-Aldrich, ST Louis, MO, United States) with 3% (v/v) fetal calf serum (FCS) (HyClone, Logan, UT, United States). *H. pylori* suspension was inoculated in each well to obtain a final concentration of approximately 5 × 10^5^ CFU × well. The 96-well microtiter plates were incubated at 37°C under microaerophilic conditions and examined after 72 h of incubation. The MBC was determined as the lowest concentration of SUNCs able to kill 99.9% of the starting inoculum. Aliquots (100 μl) of each suspension without visible growth were spotted on Columbia agar plates and incubated at 37°C for 3–5 days under microaerophilic conditions. The bactericidal activity of SUNCs was also evaluated by killing curves. The reference strain (ATCC 43504) and a clinical isolate (strain 23) susceptible to metronidazole, clarithromycin, and amoxicillin were used in the liquid culture study in the presence of different SUNCs at 0.5 (0.5×), 1 (1×), and 2 times (2×) the MIC. After 0, 24, and 48 h of incubation, the number of colony-forming units (CFU) was assessed by plating serial dilution of the samples onto Columbia agar plates. The rate and extent of killing were expressed as viable count (log_10_ CFU/ml) against time.

### Combination Effect of Metronidazole and Clarithromycin With SUNCs

The combination effect of metronidazole and clarithromycin and SUNCs was determined by checkerboard assay and evaluated using fractional inhibitory concentration index (FICI). The inoculum size and the culture conditions were the same as those used for MIC determination. The FICI was calculated from the fractional inhibitory concentration (FIC) values of test compounds and antimicrobials. The FICI ≤ 0.5, >4.0, and >0.5–4 were defined as synergistic, antagonist, and non-synergistic or additive, respectively (Doerne, 2014).

### Evaluation of the Minimum Biofilm Eradication Concentration

The biofilms were developed as previously described (Grande et al., 2011). The antibiofilm effect of each sample was determined by the evaluation of Minimum Biofilm Eradication Concentration (MBEC). The MBEC was defined as the lowest concentration of SUNCs that completely eradicated bacterial biofilm formed in 96-well flat-bottom polystyrene microtiter plates. Briefly, *H. pylori* ATCC 43504, *H. pylori* 23, *H. pylori* 110R, and *H. pylori* F40/499 were grown in BB plus 2% FCS and 0.3% (*w*/*v*) glucose (Sigma-Aldrich). The ON broth cultures were diluted till OD_600_ = 0.1 and diluted 1:10 to reach 10^6^ CFU/ml. Two hundred microliters of the diluted broth cultures was inoculated into 96-well flat-bottom polystyrene microtiter plates (Eppendorf, Hamburg, Germany) and incubated at 37°C for 48 h under static conditions as previously described (Ronci et al., 2019). At the end of incubation, the biofilms were rinsed in phosphate-buffered saline (PBS) and SUNCs were added to the mature biofilms at concentrations corresponding to 1× MIC, 2× MIC, 3× MIC, 4× MIC, and 6× MIC. Controls consisting of (i) *H. pylori* biofilms without the addition of SUNCs, (ii) BB plus 0.3% (*w*/*v*) glucose and 2% of FCS and SUNCs, and (iii) just medium plus 0.3% (*w*/*v*) glucose and 2% of FCS were inserted in the experiments. The plates were then incubated at 37°C for 24 h under static conditions. The inhibitory effect was measured using AlamarBlue (AB) assay (Thermo Fisher Scientific, Waltham, MA, United States) and CFU count. Three independent experiments were performed in triplicate.

### AB Biofilm Eradication Assay

The biofilms treated with different concentrations of SUNCs (0.32 mg/L, 0.64 mg/L, 0.96 mg/L, 1.28 mg/L, and 1.92 mg/L) were subsequently rinsed with PBS, and AB was added following the manufacturer’s instructions. The plates were incubated for 4 h at 37°C and the absorbance was read. The percentage reduction of AB in the treated and non-treated samples was calculated using the formula indicated by the manufacturer. The AB MBEC was defined as the lowest concentration of the test sample resulting in ≤50% reduction of AB and a purplish/blue well, 4 h after the addition of the AB as previously demonstrated for other microorganisms (Zengin et al., 2018). Three independent experiments were performed in quadruplicate.

### Cell Viability Evaluation Through CFU Count and Live/Dead Staining

Colony-forming unit enumeration was performed to evaluate bacterial cell viability in the biofilm phenotype after the addition of AB solution. One hundred microliters of sample solutions taken from the MBEC wells were used for CFU count. Serial dilutions of the stock were performed in PBS (pH 7.2), plated on Chocolate Agar (CA) and incubated at 37°C under microaerophilic conditions, for 3–5 days. The antibiofilm activity of SUNCs was confirmed by using the Live/Dead *BacLight* bacterial viability kit (Life Technologies, Carlsbad, CA, United States) according to the manufacturer’s instructions, followed by fluorescence microscopy analysis as previously reported (Zengin et al., 2018).

### MTT Assay

Cell toxicity was evaluated using the MTT (3-(4,5-dimethylthiazol-2-yl)-2,5-diphenyl-2*H*-tetrazolium bromide) assay (Mossman, 1983). Briefly, AGS cells seeded in a 96-well microtiter plates at 10^5^ cell/ml were treated with serial dilution of test compounds for 24 h, using DMSO (Sigma-Aldrich) as control. At the end of incubation, MTT solution (Sigma) diluted in PBS (5 mg/ml) was added, and incubation continued for an additional 3 h at 37°C in the dark. The plates were then read on a microplate reader (Synergy IV, BioTek Instruments, United States) using a test wavelength of 550 nm and a reference wavelength of 650 nm. The optical density at 650 nm (OD 650) was subtracted from the OD 550 to eliminate non-specific background. The effect was expressed as percentage of the optical density measured in cultures that did not receive SUNCs (100% viability).

### Statistical Analysis

The differences in the means of the results between untreated and treated *H. pylori* were analyzed by Student’s *t* test. The probability value of *p* ≤ 0.05 was considered significantly different.

## Results

### Characterization of SUNCs

**FIGURE 1:**
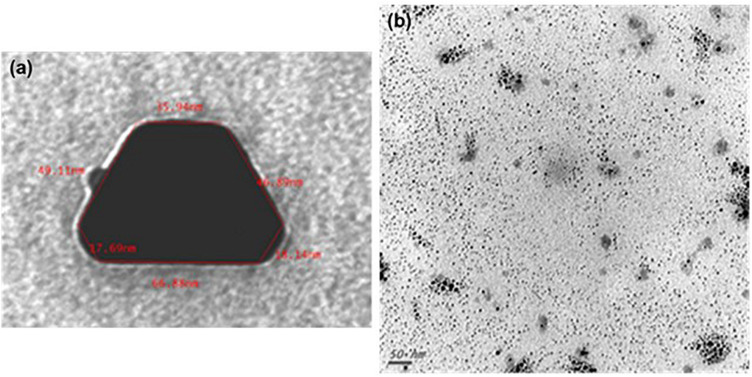
Transmission electron microscopy of SUNCs. SUNCs were electrochemically synthesized in ultrapure water. Large non-spherical nanocluster before filtration. Magnification: 250,000× **(a)**; ultra-nanoclusters after filtration. A drop of 1:5 diluted stock solution of SUNCs was allowed to evaporate onto 300 mesh formvar-coated nickel grids, and then TEM image was taken at 75 kV by a ZEISS 109 microscope. Scale bar: 50 nm. Magnification: 85,000× **(b)**.

**FIGURE 2:**
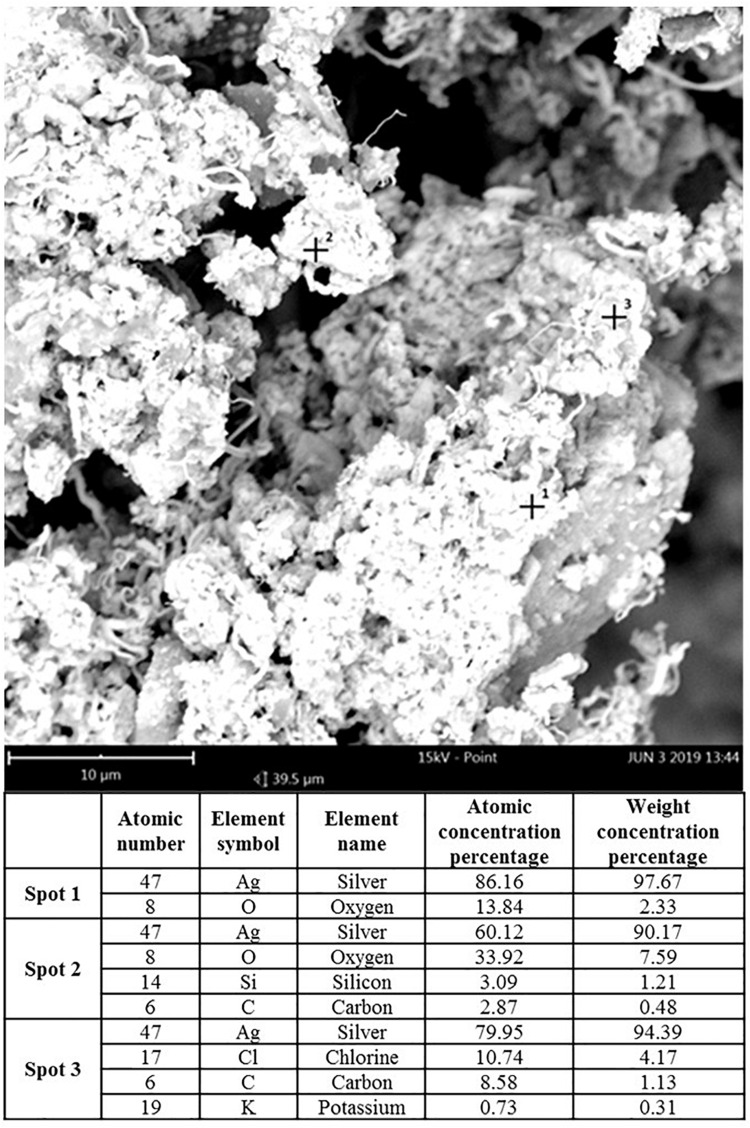
Scanning electron microscopy analysis of SUNCs. Magnification: 85,000× (scale bar: 10 μm–15 kV). Spots 1, 2, and 3 revealed different distribution of elements in the sample-different phases according to the SEM elemental analysis.

Transmission electron microscopy and ISE analyses indicated that SUNCs were characterized by a non-spherical shape (Figure 1) at concentration of 20.9 mg/L (yellow solution, pH 7–8). Statistical evaluation of TEM images demonstrated that the average size of the nanoclusters is 1.83 nm ± 1.57 (5.366 points, with min and max values of 1.15 and 13.75 nm, respectively). The prevalent abundance of the silver metal (Ag°) in the formulation was assessed by the typical (UV-Vis) plasmonic resonance spectrum (λ_max_ at 410 nm) and by SEM. Analysis of SEM images (Figure 2) indicated the presence of regions with different elemental composition. Three different regions (spots 1, 2, and 3) showed the presence of different elements: Ag, O, Si, C, Cl, and K. Ag° was predominant in all the evaluated areas. It is interesting to note that for spots 1 and 2, the second abundant element was oxygen, suggesting the possibility for Ag of being present at different oxidation states. The plasmonic spectrum showed no substantially variation in 0.01 M acetate buffer (Axson et al., 2015; D’souza et al., 2015), indicating that pH 3.5 did not affect the stability and the aggregation state of SUNCs. Only after nitric acid treatment (pH < 1) for 1 h at 60°C was a complete disappearance of the plasmonic spectrum, without a shift of the λ_max_, obtained. It is very likely that these drastic conditions can induce the complete oxidation of metal silver in Ag^+^ without macroaggregate formation (Ertürk, 2019).

### Determination of MICs and MBCs

The antibacterial activity of SUNCs was determined against nine *H. pylori* strains. As shown in Table 1, SUNCs showed antibacterial activity at MIC value ranging from 0.16 to 0.33 mg/L. No amoxicillin-resistant strain was available for this study. In addition, the bactericidal activity was performed by the microdilution method. MBC values were 1- to 2-fold MIC concentrations, demonstrating the bactericidal effects of SUNCs against *H. pylori* strains with different antibiotic susceptibility.

**TABLE 1:** Anti-*Helicobacter pylori* activity of SUNCs determined by the microdilution assay.

*H. pylori* strains	SUNCs MIC/MBC (mg/L)	MBC/MIC	Antimicrobial susceptibility (MIC mg/L)	References
Ro1	0.16/0.32	2	MNZ 1; CLR 64; AMX 0.016	Zengin et al., 2018
ATCC 43504	0.32/0.32	1	MNZ 128; CLR 0.064; AMX 0.032	
23	0.32/0.32	1	MNZ 1; CLR 0.064; AMX 0.032	Sisto et al., 2000
110R	0.32/0.32	1	MNZ 128; CLR 0.03; AMX 0.016	Brenciaglia et al., 1996
F1	0.16/0.16	1	MNZ 2; CLR 4; AMX 0.064	Zengin et al., 2018
190	0.16/0.32	2	MNZ 1; CLR 0.032; AMX 0.032	Sisto et al., 2000
F40/499	0.32/0.32	1	MNZ 32; CLR 8; AMX 0.016	Zengin et al., 2018
F40/442	0.32/0.32	1	MNZ 64; CLR 0.015; AMX 0.015	Zengin et al., 2018
F34/497	0.32/0.32	1	MNZ 128; CLR 4; AMX 0.064	Zengin et al., 2018

*MNZ, metronidazole; CLR, clarithromycin; AMX, amoxicillin.*

### Killing Kinetics

**FIGURE 3:**
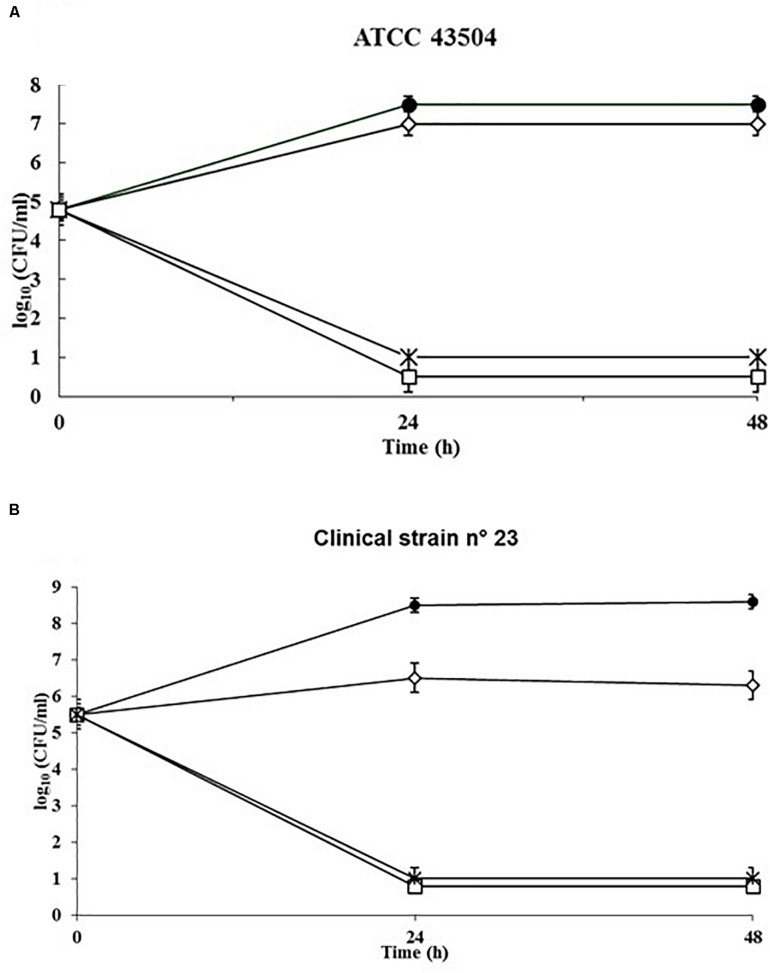
Kinetics of the killing activity of SUNCs against *H. pylori* ATCC 43504 and the clinical isolate *H. pylori* 23 by SUNCs. Antibacterial activity was evaluated in RPMI Megacell with 3% FCS in the presence or absence of the SUNCs concentrations for different time points. The MIC value of *H. pylori* ATCC 43504 **(A)** and clinical *H. pylori* 23 **(B)** was 0.33 mg/L. The data are expressed as mean CFU ± SD recovered from three different experiments performed in duplicate. Symbols: filled circle, control; open diamond, 0.5× MIC; asterisk, 1× MIC; open square, 2× MIC.

To evaluate the rate of killing of *H. pylori* by SUNCs, a kinetic study was performed against the reference strain ATCC 43504 (Figure 3A) and the clinical isolate 23 (Figure 3B). The control kinetics showed an exponential growth phase for the first 24 h, and a stationary phase for the following 24 h. SUNCs caused a ∼7-log_10_ decrease in cell count at their MIC concentration (0.33 mg/L) and the total killing within 24 h for both strains. The limit of detection was 10 CFU/ml.

### Combination Effect of Antibiotics With SUNCs

Metronidazole and clarithromycin are the antimicrobials often used as first-line treatment against *H. pylori* infections. A checkerboard titration assay against two strains of *H. pylori* was performed to verify a possible synergistic effect between SUNCs and both antimicrobial drugs. The reference strain (ATCC 43504) resistant to metronidazole (MIC > 16 mg/L) and strain 23 sensitive to clarithromycin, amoxicillin, and metronidazole were selected for this assay. SUNCs showed a value very close to synergism (FICI 0.51) with clarithromycin on both strains (Table 2), and with metronidazole on the reference strain (FICI 0.55); a synergistic effect was obtained on clinical *H. pylori* 23 (FICI 0.42) in combination with metronidazole. These results are encouraging for a possible synergistic interaction.

**TABLE 2:** Checkerboard titration assay of SUNCs in combination with MNZ and CLR.

FIC index	MNZ	FICI	CLR	FICI
≤0.5 (synergy)	Strain 23	0.42	Strain 23	0.51
0.5 < FIC ≤ 4 (not synergistic/additive interaction)	ATCC 43504	0.55	ATCC 43504	0.51

### Evaluation of MBEC

**FIGURE 4:**
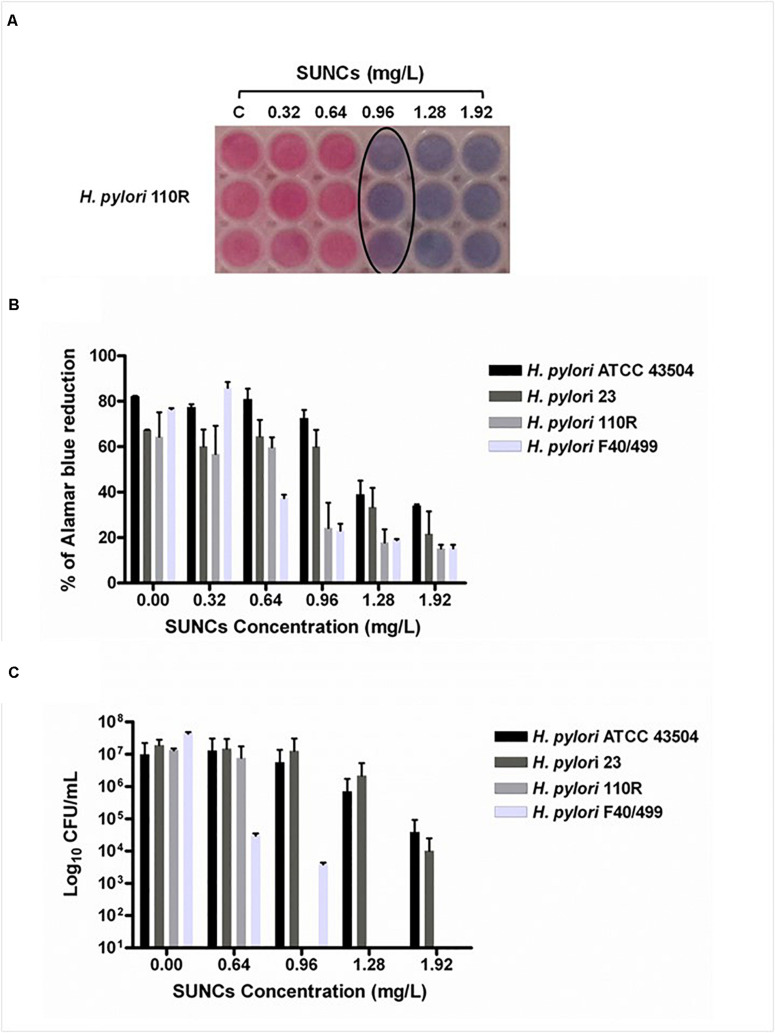
The determination of the Minimum Biofilm Eradication Concentration (MBEC) against the *H. pylori* biofilm was evaluated by using the AB assay and the CFU counting. Representative image of colorimetric MBEC evaluation by using AB. The black circle indicates the MBEC at 0.96 mg/L against *H. pylori* 110R **(A)**. The plot displays the percent reduction of AB in *H. pylori* biofilms at different concentrations of SUNCs compared to the corresponding untreated biofilms **(B)**. CFU count of SUNCs-treated and untreated biofilms **(C)**. Data are presented as the mean of three replicates of three independent experiments. Controls correspond to 0 mg/L.

**FIGURE 5:**
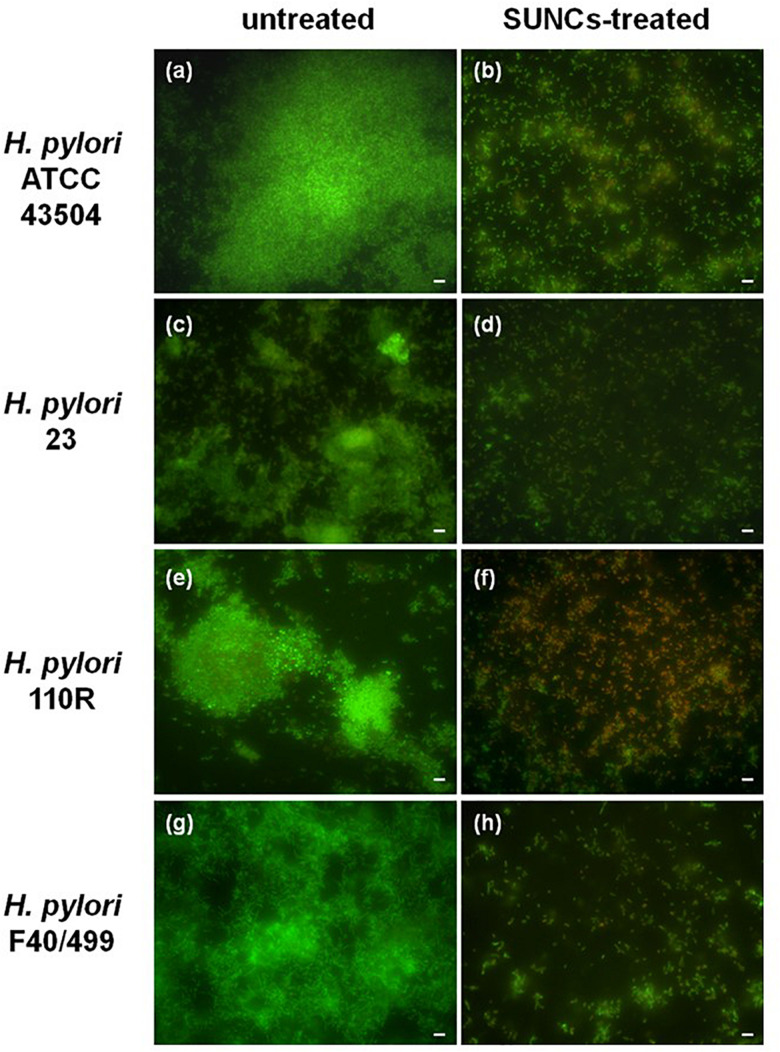
Representative *H. pylori* biofilms stained with Live/Dead kit and analyzed using fluorescence microscopy. The green fluorescence indicates the live cells, whereas the red fluorescence indicates the dead cells or cells with a damaged cell wall. Panels **(a,c,e,g)** show the untreated *H. pylori* biofilms, while panels **(b,d,f,h)** show *H. pylori* biofilms treated with SUNCs at MBEC concentrations of 1.28 mg/L for *H. pylori* strains ATCC 43504 and 23, 0.96 mg/L for 110 R, and 0.64 mg/L for F40/499. Scale bar: 5 μm.

The capability of SUNCs to eradicate the biofilm developed by *H. pylori* after 2 days of incubation was determined by using AB assay, CFU counting, and Live/Dead staining followed by fluorescence microscopy analysis. The anti-biofilm effect of SUNCs was evaluated against 3 *H. pylori* clinical strains characterized by a different antimicrobial susceptibility pattern and versus the reference strain. SUNCs showed a MBEC value of 1.28 mg/L versus *H. pylori* ATCC 43504 and *H. pylori* 23, a MBEC value of 0.96 mg/L against *H. pylori* 110R and 0.64 mg/L against *H. pylori* F40/499. The MBEC was determined by using the AB assay based on resazurin reduction by viable cells, in resorufin, a highly fluorescent red/purple compound (Figures 4A,B). The efficacy of SUNCs on *H. pylori* biofilm eradication was confirmed by both the reduction of CFU counts (Figure 4C) and fluorescence microscopy analysis (Figure 5 and Supplementary Figure S2). SUNCs caused a ∼1-log_10_ decrease in cell count at 1.28 mg/L for *H. pylori* ATCC 43504 and *H. pylori* 23 and a ∼7-log_10_ and ∼4-log_10_ decrease in cell count at 0.64 mg/L for *H. pylori* 110R and *H. pylori* F40/499, respectively. The fluorescence microscopy has been used as a qualitative analysis. The images showed a visible disaggregation of the biofilm in all SUNCs-treated strains confirming the CFU reduction (Figure 5 and Supplementary Figure S2). In particular, the addition of SUNCs at concentrations corresponding to the MBEC of the tested strains displayed a detachment of the biofilm or the presence of few cells, many of which were dead (red fluorescence), attached to the surface of the plates. We speculate that dead cells tend to detach from the surface returning to the planktonic phase. The cells that remain adhered are typically live cells, tenaciously attached and dead cells still trapped in the EPS matrix (Figures 5B,D,F,H and Supplementary Figures S2B,D,F,H). This hypothesis was confirmed by the quantitative analysis based on the CFU counts and AB assay. On the contrary, the untreated biofilm was characterized by large aggregates of living cells as indicated by the green fluorescence (Figures 5A,C,E,G and Supplementary Figures S2A,C,E,G).

### Effects of SUNCs Cells Viability

**FIGURE 6:**
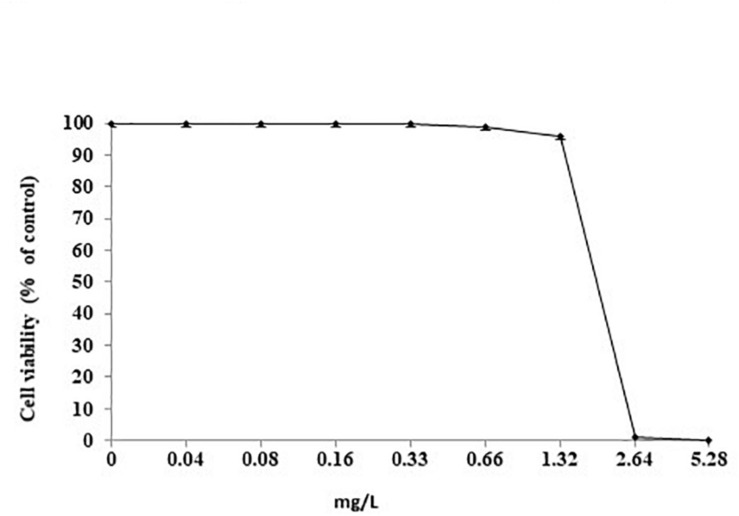
Cell viability after treatment with SUNCs by MTT assay. AGS cells were incubated for 24 h with different doses of SUNCs in F12 medium. The effect is expressed as percentage of the optical density (OD) ± SD measured in non-treated cultures (100% viability).

The effect of increasing concentrations of SUNCs (from 0.04 to 5.28 mg/L) was evaluated by MTT assay (Figure 6). SUNCs at concentrations of 0.04, 0.08, 0.16, and 0.33 mg/L resulted in 100% viability in AGS cells. A dose-dependent curve was obtained at 0.66 and 1.32 mg/L with 99% and 96% viability, respectively. At 2.64 mg/L, a dramatic decrease of viability was obtained (1% viability). For this reason, it was not possible to calculate the IC_50_. In order to evaluate the effects of SUNCs on different cell lines, MTT assay on HaCaT and HMEC was also performed. A dose-dependent toxicity was observed for HMEC with IC_50_ of 2.84 mg/L. HaCaT cells showed excellent viability also after the treatment at SUNCs concentration of 5.28 mg/L (details in Supplementary Material and Supplementary Figure S3).

## Discussion

The antimicrobial effect of different Ag^+^ ion formulations against *H. pylori* has already been demonstrated *in vivo* and *in vitro*, but few studies on the efficacy of AgNPs are present in the literature do date. Very recently, a study about the effect of biocompatible AgNPs against *H. pylori* MH179988 has been published (Saravanakumar et al., 2019). The authors reported a MIC of 18.14 mg/L, which is 8 to 10 times higher than that found in the present study, highlighting the excellent bactericidal efficacy of SUNCs. Due to the fact that many studies also aimed at analyzing the toxic effects of silver ions on human cells and in *in vivo* systems (acute exposure, bioavailability, and bioaccumulation) (Stensberg et al., 2011) and that the results often correlated the AgNP size and shape to toxicity and ADME properties, we proposed the synthesis and characterization of SUNCs endowed with dimensions <5 nm. The SEM analysis indicates the presence, at the clusters’ surface, of different Ag oxidative states whose higher reactivity might explain the present results, although further investigations are needed to better assess the peculiar chemical–physical properties of these SUNCs.

The new silver formulation showed to be very effective against different *H. pylori* strains tested with also a synergistic/additive effect when combined with MNZ and CLR. In comparison with MNZ alone, SUNCs were found to be active at their MIC value approximately three- or sixfold lower than that of MNZ-sensitive strains (MIC MNZ = 1 mg/L). In the AGS cell model, the MTT assay showed that SUNCs were toxic at 2.62 mg/L, ∼4 and 3 log_2_ dilutions higher than their MIC and/or MBC values, respectively. These results clearly corroborated the limited cell toxicity of our SUNCs with respect to commercially available AgNPs characterized by greater dimensions, as reported in the literature (Duran et al., 2010).

It has been widely demonstrated that *H. pylori* develops biofilm both *in vitro* and *in vivo* (Cellini et al., 2005, 2008; Carron et al., 2006; Grande et al., 2015). As reported by Koo et al. (2017) the biofilm, initially defined as the “arcane behavior of bacterial populations,” is considered today as a “principal virulence factor in many localized chronic infections.” Biofilm infections commonly recur after long periods of clinical quiescence, and the key role of the EPS biofilm matrix in ensuring antimicrobial tolerance to biofilms is widely demonstrated (Koo et al., 2017).

Among the common features of microbial biofilms, there is the adherence. In fact, microorganisms adhere to both abiotic materials such as plastic, metals, ceramics, etc., and biotic surfaces such as tooth enamel, bone, skin, intestinal, vaginal mucosa, and connective tissues, but also gastric mucosa as demonstrated by **Carron et al. (2006)** who showed *H. pylori* biofilm on human gastric mucosa. The multifactorial nature of biofilm development and drug tolerance dictates important choices in the use of conventional antimicrobial drugs and dictates the need for multi-target or combinatorial therapies, particularly for *H. pylori* infections often associated with multidrug-resistant strains toward antibiotics commonly used in therapy such as clarithromycin. Therefore, the identification of alternative therapies also associated with the use of antibiotics commonly administered in clinical practice is necessary. The microbial community of the biofilm may develop tolerance to the antibiotics using different mechanisms such as the bacterial conversion from the culturable status into a VBNC status (Sisto et al., 2000), or the development of molecular pathways that promote the bacterial persistence and survival (Flemming et al., 2016). The efficacy of SUNCs has been tested to evaluate its possible application for the treatment of *H. pylori* infections associated with the biofilm development. The capability of SUNCs to eradicate a 2-day preformed biofilm is probably due to nanoparticle penetration through the biofilm EPS matrix, where they are less susceptible to modifications compared to common drugs (Dakal et al., 2016). On the other hand, **Gurunathan et al. (2015)** demonstrated that AgNPs, with an average size of 20 nm, caused dose-dependent decrease in cell viability and biofilm formation as well as DNA fragmentation in *H. pylori* and *H****elicobacter*** *felis*. Moreover, the data obtained showed the capability of SUNCs to kill planktonic cells and to eradicate the biofilms developed by all strains analyzed, regardless of their pattern of sensitivity to antimicrobial drugs. The fluorescence microscopy images show the presence of few cells adhered to the plate surface in the SUNCs-treated samples, suggesting that the SUNCs disrupt the biofilm. We hypothesize that SUNCs induce cell death, promoting the cell detachment from the surface. The few remaining cells, in fact, are live cells tenaciously attached to the surface, or dead cells trapped in the EPS matrix. The use of SUNCs as antimicrobial could reduce the drug resistance; therefore, they could be used in combination with antimicrobial drugs, leading also to a decrease in human cell toxicity due to the reduction of the dosage. SUNCs could also be considered a carrier for drug delivery as previously reported (Sharvil et al., 2009). Furthermore, SUNCs at 1.32 mg/L killed 100% of bacteria with 100% viability of AGS cells. Better results were obtained with HaCaT cells that did not show any toxic effect at all concentrations tested (Supplementary Figure S3). Many studies report the low toxicity of AgNPs on different cell lines (Ivask et al., 2014; Kanipandian et al., 2014; Chhibber et al., 2017). Although *in vitro*, these results are very encouraging and suggest differences in the toxicity mechanisms of SUNCs in different biological systems. In addition, we showed that SUNCs are about 10-fold less toxic in mammalian than in bacterial cells, suggesting different toxic mechanisms toward different biological systems. A recent review (Ullah Khan et al., 2018) summarized the toxic effects of **AgNP**s against bacteria, humans, fungi, viruses, and protozoa. In general, low amount of **AgNP**s has excessive potential against microorganisms, while at higher concentrations **(>**10** μ**M), they are toxic to mammals. Conversely, other studies report that humans can tolerate the assumption up to 16 mg with low adverse effects (Kim et al., 2012, 2013; Amin et al., 2014), or propose nanosilver as a pharmaceutical agent that is non-toxic for humans (Lansdown, 2006). These findings, together with the results reported here, strongly support the idea that a possible SUNCs therapy *in vivo* against *H. pylori* infection could be characterized by limited unwanted side effects (Ottoni et al., 2019; Rodrigues et al., 2019). Indeed, the absorption of **AgNP**s and their toxicity on human intestinal cells have been monitored by Böhmert et al. (2014). The results suggest that **AgNP**s may overcome the gastrointestinal juices in their particulate form without forming large quantities of aggregates. Consequently, the authors presumed that the particles can reach the intestinal epithelial cells after ingestion with only a slight reduction in their cytotoxic potential. Furthermore, a recent review (McClements and Xiao, 2017) summarized the factors affecting the gastrointestinal fate and toxicity of organic and inorganic food-grade nanoparticles. Animal studies (Kim et al., 2008; [Bibr B20][Bibr B27]) corroborated that **AgNP**s can be absorbed by the gastrointestinal tract into the systemic circulation, and then be distributed throughout various organs. However, only a small fraction **(<**1%) of ingested **AgNP**s typically accumulate in tissues, which suggests that the majority of them were excreted in the feces or urine (Hendrickson et al., 2016). At the levels used in that study (2000 and 250 mg/kg body weight for single and multiple doses, respectively), no toxicity of the **AgNP**s was found after oral gavage. Another rat feeding study reported no major toxic effects of ingestion of **AgNP**s over a 28-day period (30, 300, and 1000 mg/kg day), but that there was some slight liver damage at the highest levels used (Kim et al., 2008). These results could be a good starting point in order to relate the concentrations that should be delivered *in vivo* with the effective concentrations observed here *in vitro* and, in the future, *in vivo* pharmacokinetic assays will be performed to assess the clinical efficacy and toxicity in a more complex model. To the best of our knowledge, this is the first study reporting the antimicrobial and anti-biofilm effects against *H. pylori* of SUNCs with dimensions smaller than 5 nm and synthesized in UPW. SUNCs could represent a novel strategy for the treatment of *H. pylori* infections especially in cases of multidrug resistance or biofilm-producing strains. Although the strains used in this study have different antibiotic susceptibility profiles, further investigations on a greater collection of isolates with established diverse genetic backgrounds are also to be considered. Although the synergistic activity of SUNCs in association with well-established antimicrobial drugs was performed versus two strains, the data obtained suggest the potential use of SUNCs in combination therapy with traditional antibiotics in order to improve the management of *H. pylori* infection. Further, *in vivo* pharmacokinetic assays will be performed to assess the clinical efficacy and toxicity in a more complex model.

## Data Availability Statement

All datasets generated for this study are included in the article/Supplementary Material.

## Author Contributions

FS and RG designed, interpreted the data, and wrote the article. FS and VP performed the experimental work and acquisition of data. MR, SC, and LS synthesized the nanoparticles and performed the characterization and SEM/TEM analyses. GM analyzed the data and performed the statistical analysis. RM and AA critically revised the work and the final manuscript. RG funded the project. All authors contributed to the article and approved the submitted version.

## Conflict of Interest

LS is the author of a pending European Patent (EP-18181873.3). The remaining authors declare that the research was conducted in the absence of any commercial or financial relationships that could be construed as a potential conflict of interest.
