# Supplementary material for: Antimicrobial and Antibiofilm Activities of New Synthesized Silver Ultra-NanoClusters (SUNCs) Against Helicobacter pylori
Source: Front Microbiol. 2020 Jul 31;11:1705. doi: 10.3389/fmicb.2020.01705 (PMC7411087; doi:10.3389/fmicb.2020.01705)
Supplement: Supplementary file 1 [file Data_Sheet_1.pdf]

Supplementary material

Antimicrobial and antibiofilm activities of new synthesized Silver Ultra-NanoClusters (SUNCs) against *Helicobacter pylori*

Rossella Grande[1,4§], Francesca Sisto[2,§,*], Valentina Puca[3,4], Simone Carradori[1], Maurizio Ronci[1,4], Antonio Aceto[5], Raffaella Muraro[5], Gabriella Mincione[5] and Luca Scotti[5]

[1]Department of Pharmacy, "G. d'Annunzio" University of Chieti-Pescara, Chieti, Italy

[2]Department of Biomedical, Surgical and Dental Sciences, University of Milan, Milan, Italy

[3]Department of Medicine and Aging Science, "G. d'Annunzio" University of Chieti-Pescara, Chieti, Italy

[4]Center for Advanced Studies and Technology (CAST), "G. d'Annunzio" University of Chieti-Pescara, Chieti, Italy

[5]Department of Medical, Oral, and Biotechnological Sciences, "G. d'Annunzio" University of Chieti-Pescara, Chieti, Italy

[§]**These authors contributed equally to this work**.

[*]Francesca Sisto, Department of Biomedical, Surgical and Dental Sciences, University of Milan, Via Pascal 36, 20133 Milan, Italy. Phone 0039 2 250315064; Fax 0039 2 50315068; E-mail: francesca.sisto@unimi.it

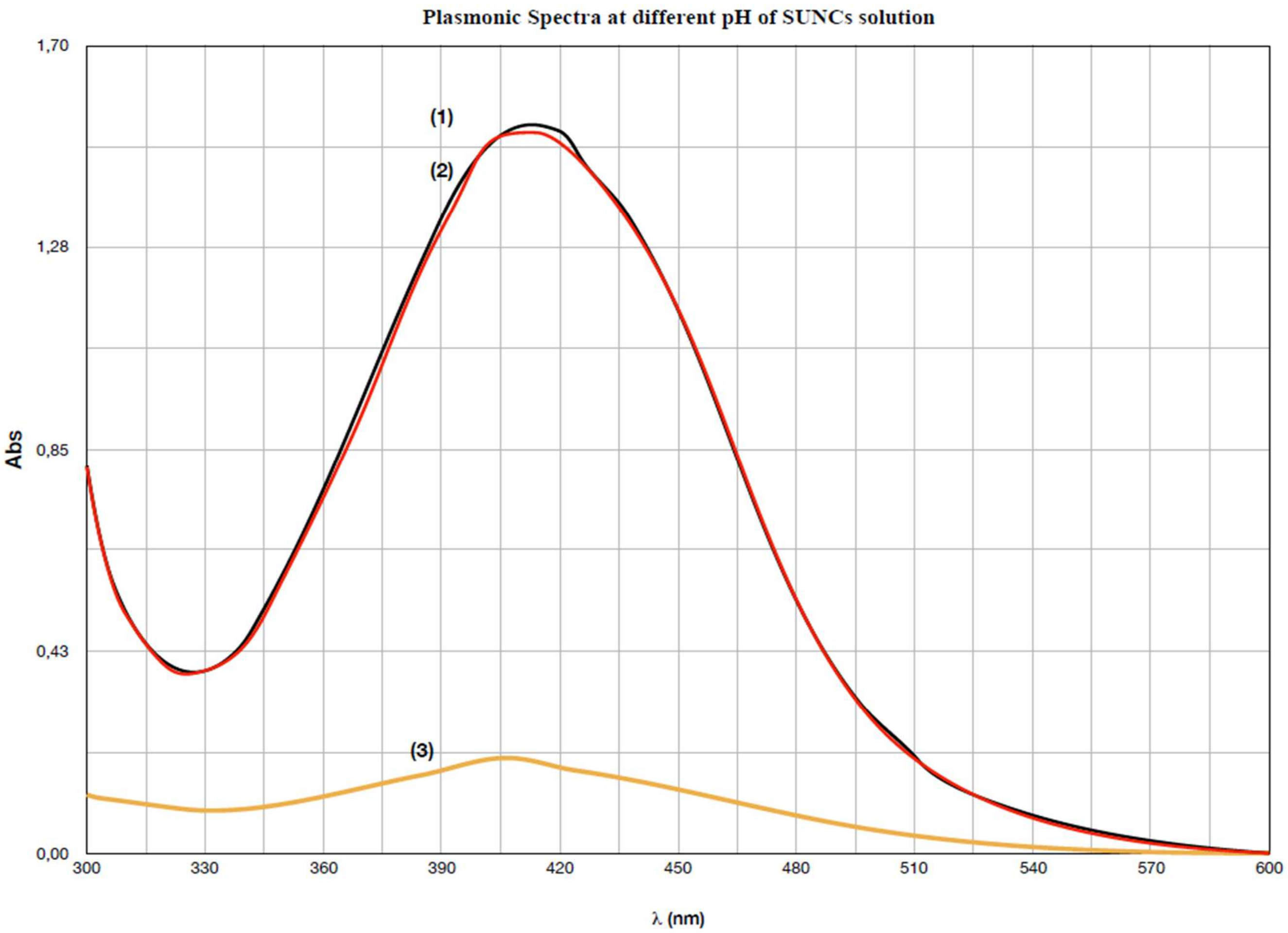

Figure S1. (1) UV spectrum of SUNCs solution (25 ppm - pH 7.5 in UP-water); (2) UV spectrum of SUNCs after 1 h of incubation at pH 3.5 in 0.01 M acetate buffer; (3) UV spectrum of SUNCs solution after nitric acid treatment (0.15 M, pH<1) for 10 min at 25 °C. The complete disappearance of the plasmonic spectrum was obtained after 60 min treatment with nitric acid (0.24 M, pH<1) at 60 °C. All spectra were recorded at different pHs with a double beam spectrophotometer (Jasco 7800, Japan), normalised at the same SUNCs concentration after blank (buffer without SUNCs) subtraction.

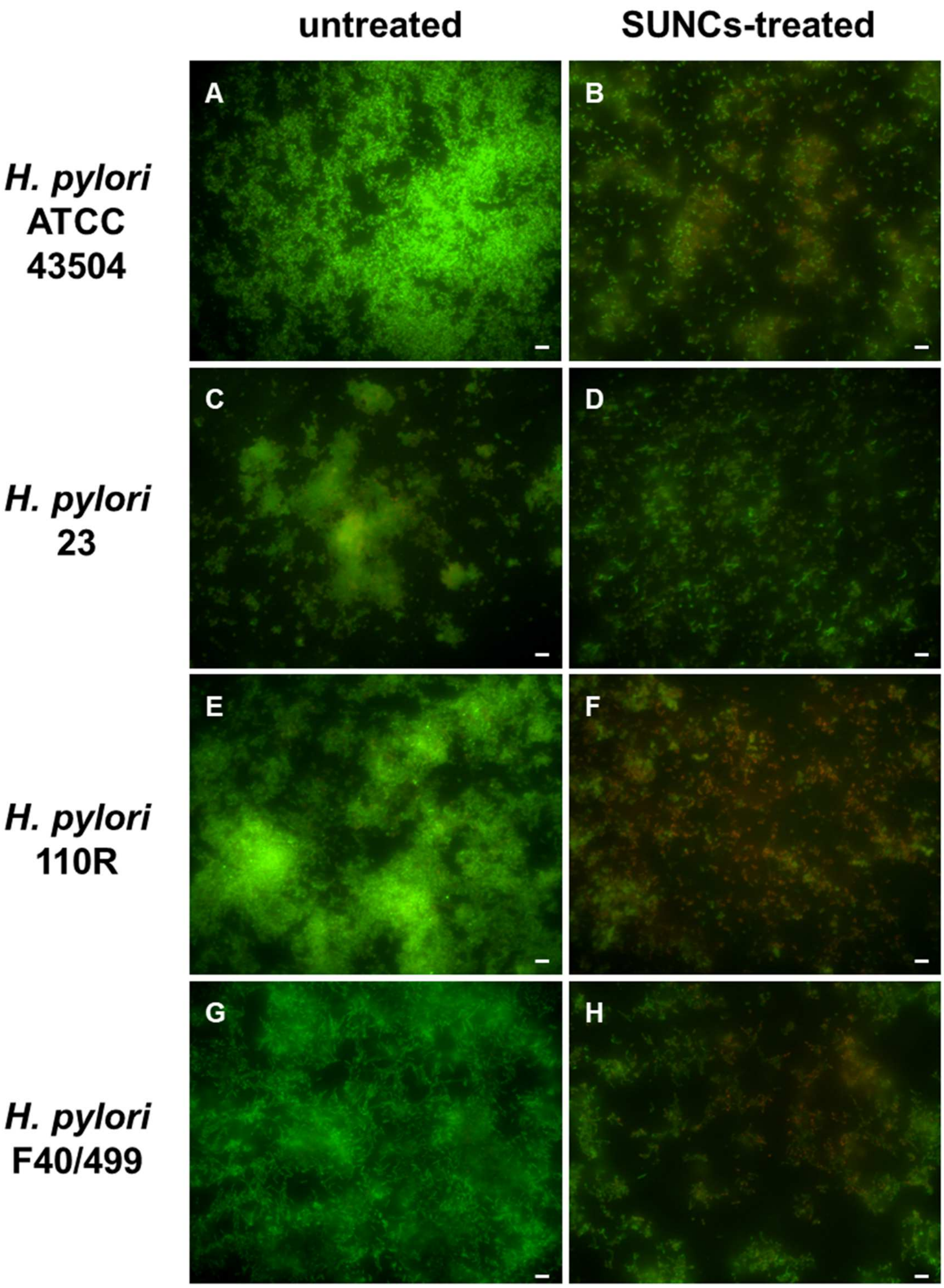

Figure S2. Additional representative *H. pylori* biofilms images stained with Live/Dead kit and analysed using fluorescence microscopy. The green fluorescence indicates the live cells, whereas the red fluorescence indicates the dead cells or cells with a damaged cell wall. Panels (A), (C), (E), (G) show the untreated *H. pylori* biofilms, while panels (B), (D), (F), and (H) show *H. pylori* biofilms treated with SUNCs at MBEC concentration of 1.28 mg/L for *H. pylori* strains ATCC 43504 and 23; 0.96 mg/L for 110 R, and 0.64 mg/L for F40/499. Scale bar: 5 µm.

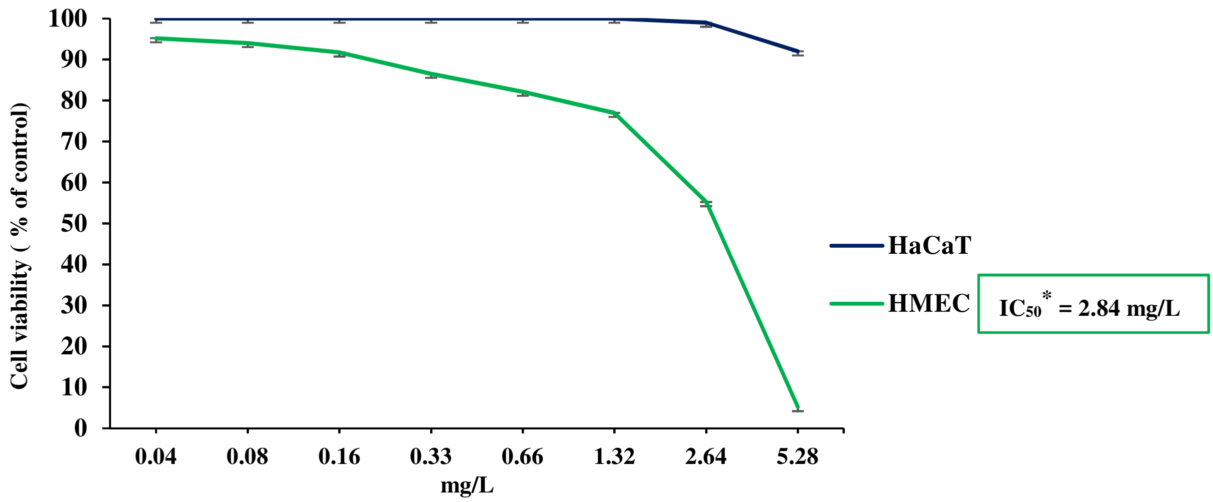

Figure S3. Cell viability after treatment with SUNCs by MTT assay. HaCaT and HMEC-1 cells were incubated for 24 h with different doses of SUNCs in DMEM high glucose and MCDB131, respectively. The effect is expressed as percentage of the optical density (OD) ± SD measured in non-treated cultures (100% viability).

*Reed, L. J. & Muench, H. A simple method of estimating fifty percent endpoints. (1938). *Am J Hyg*. 27, 493–497.

Supplementary cell culture

HaCaT cells were cultured in a 5% CO_2 atmosphere in DMEM high glucose (HyClone, Logan, UT) with 10% (*v/v*) decomplemented fetal bovine serum (FBS); HMEC were cultured in MCDB131 (Thermo Fisher Scientific, Waltham, MA, USA) with 10 ng/mL Epidermal Growth Factor (EGF) (Milteny Biotec, USA), 1 µg/mL Hydrocortisone (Sigma Aldrich), and 10% FBS in a 5% CO_2 incubator.
